# Phylogeny and maternal donors of *Elytrigia* Desv. sensu lato (Triticeae; Poaceae) inferred from nuclear internal-transcribed spacer and *trn*L-F sequences

**DOI:** 10.1186/s12870-017-1163-7

**Published:** 2017-11-21

**Authors:** Yan Yang, Xing Fan, Long Wang, Hai-Qin Zhang, Li-Na Sha, Yi Wang, Hou-Yang Kang, Jian Zeng, Xiao-Fang Yu, Yong-Hong Zhou

**Affiliations:** 1 Triticeae Research Institute, Sichuan Agricultural University, Wenjiang, 611130 Chengdu, Sichuan People’s Republic of China; 2 College of Environmental Science and Engineering, China West Normal University, Nanchong, 637009 Sichuan People’s Republic of China; 3 College of Resources, Sichuan Agricultural University, Wenjiang, 611130 Chengdu, Sichuan People’s Republic of China; 4 College of Landscape Architecture, Sichuan Agricultural University, Wenjiang, 611130 Chengdu, Sichuan People’s Republic of China

**Keywords:** *Elytrigia* Desv., Chloroplast *trn*L-F, Nuclear ITS, Phylogeny, Maternal donor

## Abstract

**Background:**

*Elytrigia* Desv. is a genus with a varied array of morphology, cytology, ecology, and distribution in Triticeae. Classification and systematic position of *Elytrigia* remain controversial. We used nuclear internal-transcribed spacer (nrITS) sequences and chloroplast *trn*L-F region to study the relationships of phylogenetic and maternal genome donor of *Elytrigia* Desv. sensu lato.

**Results:**

(1) E, F, P, St, and W genomes bear close relationship with one another and are distant from H and Ns genomes. E^e^ and E^b^ are homoeologous. (2) In ESt genome species, E genome is the origin of diploid *Elytrigia* species with E genome, St genome is the origin of *Pseudoroegneria*. (3) Diploid species *Et. elongata* were differentiated. (4) *Et. stipifolia* and *Et. varnensis* sequences are diverse based on nrITS data. (5) *Et. lolioides* contains St and H genomes and belongs to *Elymus* s. l. (6) E genome diploid species in *Elytrigia* serve as maternal donors of E genome for *Et. nodosa* (PI547344), *Et. farcta*, *Et. pontica*, *Et. pycnantha*, *Et. scirpea*, and *Et. scythica*. At least two species act as maternal donor of allopolyploids (ESt and EStP genomes).

**Conclusions:**

Our results suggested that *Elytrigia* s. l. species contain different genomes, which should be divided into different genera. However, the genomes of *Elytrigia* species had close relationships with one another. Diploid species were differentiated, because of introgression and different geographical sources. The results also suggested that the same species and the same genomes of different species have different maternal donor. Further study of molecular biology and cytology could facilitate the evaluation of our results of phylogenetic in a more specific and accurate way.

## Background

Triticeae in Poaceae includes not only the most economically important cereal crops (wheat, barley, and rye) but also forage grasses and ecological species in grasslands. Approximately 450 Triticeae species exist worldwide [1–3]. Given the wide variety of biological mechanisms and genetic systems, this tribe represents an excellent group for research on plant systematics, genetic diversity, and speciation [4, 5].

As one of the most important perennial genera of Triticeae, *Elytrigia* Desv. includes 40 species, which are distributed in subtropical and warm temperate regions of both hemispheres [6]. *Elytrigia* Desv. was established by Desvaux [7], with *Elytrigia repens* (L.) Nevski as the type species. Morphologically, *Elytrigia* sensu lato is characterized by branched creeping rhizomes and caespitose, long anthers, lanceolate to liner glumes, lanceolate lemma, single spikelet per node, and cross-pollination, and most species were previously categorized under *Agropyron* Gaertner [1, 6, 8]. Cytogenetically, ploidy levels in *Elytrigia* s. l. vary from diploid (2n = 2× = 14) to decaploid (2n = 10× = 70) and contain E^e^, E^b^, St, ESt, StH, and NsXmStH genomes [1, 3, 8–11]. According to the proposed genomic system of classification, Löve [8] suggested that *Elytrigia* s. l. approximately includes 60 species and varieties and divided them into five genera, namely, *Pseudoroegneria* (Nevski) Á. Löve (St), *Lophopyrum* Á. Löve (E), *Thinopyrum* Á. Löve (J), *Elytrigia* (EJSt), and *Elymus* (StH). Dewey [1] considered *Elytrigia* s. l. into three independent genera: *Pseudoroegneria* (St), *Thinopyrum* (E or J), and *Elytrigia* (StX). Studies showed similarity of the E and J genomes [12–19]. Wang et al. [19] suggested that E and J should be considered as identical genomes and be distinguished from E^e^ and E^b^. With the genomic system of classification in Triticeae taxonomy and systematics and genomic constitutions of increasing species identified, the definition of *Elytrigia* becomes narrower than that of traditional *Elytrigia* s. l. and only includes all polyploidy taxa with combination of E^e^, E^b^, and St genomes [3, 9, 11]. E^e^ genome originated from *Lophopyrum elongatum* (Host) Á. Löve, E^b^ genome from *Thinoopyrum bessarabicum* (Savul. and Rayss) Á. Löve, and St genome from diploid species in *Pseudoroegneria* (Nevski) Á. Löve. However, some studies reported that *Et. repens*, a type of *Elytrigia*, is a hexaploid species with StStStStHH genomes and was renamed as *Elymus repens* [3, 11, 20, 21]. Therefore, definition, precise taxonomic ranks, and number of *Elytrigia* species remain controversial.

Polyploidization and hybridization are the two main mechanisms in plant speciation and evolution [22, 23]. The changes in the cell size, genome size, gene expression, genomic repatterning, epigenetic effects and retrotransposon activation are caused by the polyploidization and chromosome doubling [5, 22–26]. As a result of these changes, stabilization of hybrid condition and full fertility may occur. And the establishment of phenotypes in nature could be enhanced. Therefore, polyploids could adjust to match with the new ecological niches or become more competitive than parental donors [5, 23, 26, 27]. The evolution of polyploidization alone and/or the combined effects of hybridization and polyploidization may lead to complex lineages, requiring an explanation of the phylogenetic relationship [27]. Molecular genetic analysis bears significance in elucidating phylogenetic relationships and genome evolution patterns in taxa for these kinds of plant groups [27, 28]. The analysis of Molecular phylogenetic exploits DNA sequences elucidated the history of revolution and origins of species in Triticeae. This illustrates their hybridization events and parental lineages contains their formation, and identifies the polyploidization mode [29–38]. Reproducibility and simplicity represent the main qualities that make DNA sequencing a suitable choice for identification of phylogenetic relationships among taxa and genomes [28, 39]. nrITS sequences were widely applied to explain genomic and phylogenetic relationship at a low taxonomic levels [40–42] and Triticeae species containing E, H, Ns, P, St and Xm genomes in *Elymus*, *Hordeum*, *Psathyrostachys*, *Agropyron Pseudoroegneria* and *Leymus* [5, 27, 33, 40–43]. Chloroplast DNA (cpDNA) sequences, including intron of *trn*-L and intergenic spacer of *trn*H-*psb*A, *trn*L-*trn*F, and *trn*S-*trn*G, are also widely used to identify maternal donors of polyploids with extra ability to analyze phylogenetic relationships among relevant species [41, 44–47].

The present study analyzed sequence data of one ITS region of nuclear DNA and one chloroplast gene (the intergenic region of *trn*L-*trn*F) from 18 species (subspecies) in *Elytrigia* s. l. with 21 species of related genera in Triticeae. The objectives are as follows: (1) to investigate phylogenetic relationships among species in *Elytrigia* s. l. and related genera; (2) to elucidate interspecific relationships among *Elytrigia* s. l. species; (3) to study phylogenetic relationships among species with different genomes and genome combinations; and (4) to discuss putative maternal donors for ESt genome species.

## Methods

### Plant materials

This study included 18 species (subspecies) of *Elytrigia* Desv. sensu lato and 21 species (subspecies) of related genera in Triticeae. Table 1 lists names, accession numbers, genomes, origins, and GenBank accession numbers. *Bromus cartharticus* Vahl. and *Bromus tectorum* L. were used as outgroup [5, 41, 48]. Seed materials with W_6_ and PI accession numbers were carefully offered by the American National Plant Germplasm System (Pullman, Washington, USA). We gathered seed materials with Y and ZY numbers. Voucher specimens and plants were deposited at the perennial nursery and herbarium of the Triticeae Research Institute, Sichuan Agricultural University, China.

**Table 1:** Species of *Elytrigia* sensu lato and the related species used in this study

Species	Genome	Accession No.	Origin	GenBank No.		Abbr.
				*Trn*L-F	ITS	
*Elytrigia* Desv.						
*Elytrigia bessarabica* (Savul & Rayss) Dubov.	E^b^	PI531711	Ukraine	MF893171		EBES
		PI531712	Russian Federation		L36506^a^	
*Elytrigia caespitosa* (C. Koch) Nevski	E^e^St	PI547311	Russian Federation	EU139480^a^		ECAE
					MF893146	ECA1
					MF893147	ECA2
*Elytrigia elongata* (Host) Nevski	E^e^E^e^	W6 21,859	Iran	MF893172		EELO
					MF893148	EEL1
		PI531719	France		EF014249^a^	EEL2
*Elytrigia farcta* (Viv.) Holub	E^b^E^b^E^e^	PI516555	Morocco	MF893175	MF893149	EFAR
*Elytrigia geniculata* (Trin.) Nevski	StSt	PI565009	Russian Federation	MF893176		EGEN
*Elytrigia geniculata* ssp. *pruinifera* (Nevski) Tzvel.	-^b^	PI547374	Russian Federation	MF893177		EPRU
					EF014229	EPR1
					MF893150	EPR2
*Elytrigia intermedia* (Host) Nevski	E^b^E^e^St	PI401228	Iran	MF893179		EINT
		PI229917	Iran		MF893152	EIN1
		PI531725	Germany		MF893153	EIN2
*Elytrigia lolioides* (Kar. et Kir.) Nevski	-^b^	PI440059	Former Soviet Union	MF893180		ELOL
					MF893154	ELO1
					MF893155	ELO2
*Elytrigia nodosa* (Steven) Nevski	E^e^St	PI547344	Turkey	MF893173		ENO1
		PI547345	Ukraine	MF893174		ENO2
					EF014248	EN11
					JX624139^a^	EN12
*Elytrigia podperae* (Nábělek) Holub	E^b^E^b^E^e^	PI401299	Iran	MF893181	MF893156	EPOD
*Elytrigia pontica* (Podp.) Holub	-^b^	PI383583	Turkey	MF893183		EPO1
					MF893157	EP11
					AY090768^a^	EP12
		PI547313	Russian Federation	MF893182		EPO2
*Elytrigia pungens* (Pers.) Tutin	E^e^StStP	PI547268	Russian Federation	MF893189	MF893158	EPUN
*Elytrigia pycnantha* (Godr.) Á. Löve	E^e^StP	PI618742	Jonufer, Albania	MF893190		EPYC
		E6–1	Çanakkale, Turkey		GQ373272^a^	
*Elytrigia rechingeri* (Runemark) Hulub	E^b^E^e^	PI531745	Greece	MF893184	MF893159	EREC
*Elytrigia repens* (L.) Nevski	StStH	Y0814	China	MF893185		EREP
					MF893160	ERE1
					MF893161	ERE2
*Elytrigia scirpea* (K. Presl) Holub	E^e^E^e^	PI531749	Italy		MF893162	ESC1
		PI531750	Greece	MF893186		ESCI
					MF893163	ESC2
*Elytrigia scythica* (Nevski) Nevski	E^e^St	PI502271	Russian Federation	MF893187		ESCY
		PI283272	Former Soviet Union		MF893164	ES11
					MF893165	ES12
*Elytrigia varnensis* (Velen.) Holub	-^b^	PI281863	Germany	MF893188		EVAR
					MF893169	EVA1
					MF893170	EVA2
*Agropyron* Gaertn.						
*Agropyron cristatum* (L.) Gaertn	P	H10066	Xinjiang, China	AF519116^a^		ACRI
					AY740891^a^	
*Australopyrum* (Tsvelev) A. Löve						
*Australopyrum pectinatum* (Labill.) Á. Löve	W	M. Pinar 4412b	Turkey	KP723656^a^		APEC
		D3438	Australia		L36483^a^	
*Australopyrum retrofractum* (Vickery) Á. Löve	W	PI547363	Australia	EU617319^a^		ARET
					EU617249^a^	
*Australopyrum velutinum* (Nees) B. K	W	D2873–2878	Australia	AF519119^a^		AVEL
*Elymus* L.						
*Elymus canadensis* L.	StH	PI499412	China		KJ526334^a^	EC11
					KJ526335^a^	EC12
*Elymus caninus* (L.) L.	StH	PI564910	Russian		AY740897^a^	E111
					AY740898^a^	E112
*Eremopyrum* Jaub. Et Spach.						
*Eremopyrum distans*(C. Koch) Nevski	F	H5552	Iran	AF519150^a^		EDIS
		TA2229	Afghanistum		JQ360120^a^	
*Eremopyrum orientale*(L.) Jaub. Et Spach	F	H5555	Iran	AF519151^a^		EORI
*Eremopyrum triticeum*(Gaertn) Nevski	F	Y206	China		JQ360124^a^	ETRI
*Hordeum* L.						
*Hordeum bogdanii* Wilensky	H	PI531761	China	AY740789^a^		HBOG
					AY740876^a^	
*Hordeum chilense* Roem & Schult.	H	-^b^	-^b^	FN568308^a^		HCHI
		GRA1000	Chile		AJ607873^a^	
*Psathyrostachys* Nevski						
*Psathyrostachys fragilis* (Boiss.) Nevski	Ns	PI343192	Iran	AF519169^a^		PFRA
*Psathyrostachys juncea* (Fischer) Nevski	Ns	PI001163	China	EF581911^a^		PJUN
		Y2054	China		KT184655^a^	
*Psathyrostachys huashanica* Keng ex P. C. Kuo	Ns	ZY3157	China		JQ360145^a^	PHUA
*Pseudoroegneria* (Nevski) Á. Löve						
*Pseudoroegneria gracillima* (Nevski) Á. Löve	St-	PI420842	Russian Federation	MF893178	MF893151	PGRA
*Pseudoroegneria libanotica* (Hackel) D. R. Dewey	St	PI228391	Iran	AF519156^a^		PLIB
		PI228389			AY740794^a^	
*Pseudoroegneria spicata* (Pursh) Á. Löve	St	PI610986	United States	AF519158^a^		PSPI
		PI506259	United States		MF893166	PSP1
		PI563870	United States		MF893167	PSP2
*Pseudoroegneria stipifolia* (Czern. ex Nevski) Á. Löve	St	PI325181	Russian Federation	EF396989^a^		PSTI
		PI440095	Russian Federation		EU617052^a^	
*Pseudoroegneria strigosa* (M. Bieb.) Á. Löve	St	PI531752	Ukraine	EU139489^a^	MF893168	PSTR
*Pseudoroegneria strigosa* ssp. *aegilopoides* (Drobov) Á. Löve	-^b^	PI595164	China	EF396990^a^		PAEG
		W6 13,089	China		EU617075^a^	
*Pseudoroegneria tauri* (Boiss. & Bal.) Á. Löve	St	PI401323	Iran	EF396991^a^		PTAU
		PI380646	Iran		EsU617239^a^	
*Bromus catharticus* Vahl.	-^b^	-^b^	South Korea		KF713186^a^	
*Bromus tectorum* L.		-^b^	-^b^	EU036166^a^		
	-^b^	-^b^	South Korea		KF713207^a^	

^a^Previously published sequences from GenBank (http://www.ncbi.nlm.nih.gov)

^b^Information not available

### DNA extraction, amplification, and sequencing

Total genomic DNA was extracted from leaves of single plants by slight modification of Cetyltrimethyl Ammonium Bromide (CTAB) procedure [49]. nrITS sequence and chloroplast *trn*L-F sequence were amplified with primers described in Table 2. A final volume of 20 μL of mixed reagents was obtained for each polymerase chain reaction (PCR); reagents included 2× Taq PCR MasterMix (10× ExTaq polymerase buffer, 3 mmol/L MgCl_2_, 500 μmol/L deoxynucleotide, 100 mmol/L KCl, and 20 mmol/L Tris–HCl), 1 μmol/L of each primer, 20–40 ng of template DNA, and distilled deionized water. PCR reactions were performed in GeneAmp T100 Thermal Cycler (Bio-Rad Inc., USA) employing protocols listed in Table 3. PCR products were electrophoresed on 1.0% agarose gels, and purified using EZNA™ gel extraction kit (Omega, GA, USA), and were cloned into pMD-19 T vector (TaKaRa) following the instructions of manufacturer. All sequences were derived from at least 3 independent clones for diploid species, and 5–8 independent clones for allopolyploid species. Sequencing was performed from both directions by Sunbiotech Company (Beijing, China) [36].

**Table 2:** Names, sequences, and references of primers used in this study

Gene	Name of primers	Sequence of primer (5′- 3′)	Reference
nrITS	ITS4	TCCTCCGCTTATTGATATGC	Hsiao et al. (1995) [59]
	ITS-L	TCGTAACAAGGTTTCCGTAGGTG	
*trn*L-F	C	CGAAATCGGTAGACGCTACG	Mason-Gamer et al. (2002) [44]
	F	ATTTGAACTGGTGACACGAG

**Table 3:** Thermocycling conditions for amplification of genes using the PCR

Gene	Protocol
nrITS	1 cycle: 3 min 94 °C; 35 cycles: 1 min 94 °C, 1 min 52 °C, 1 min 72 °C; 1 cycle: 8 min 72 °C
*trn*L-F	1 cycle: 4 min 94 °C; 25 cycles: 40 s 94 °C, 50 s 60 °C, 2 min 72 °C; 1 cycle: 8 min 72 °C

### Phylogenetic analysis

Alignment of nrITS and *trn*L-F sequences were conducted by using Clustal W algorithm [50] with additional manual adjustment. Two data matrices with included nrITS were performed using Maximum likelihood (ML) in PAUP*4.0a (Swofford, D.L., Sinauer Associates, http://www.sinauer.com) and Bayesian inference (BI) in MrBayes version 3.1.2 [51]. Phylogenetic analyses based on *trn*L-F sequences were performed with MrBayes version 3.1.2. Evolutionary model employed for phylogenetic study was performed using Modeltest v3.7 with Akaike information criterion (AIC) [52]. Best-fit model was GTR + G for nrITS data. ML heuristic studies were carried out with 1000 random addition sequence replications and reconnection branch swapping algorithm and tree bisection [Dong 2013].

Similar to ML analysis, BI analyses of nrITS were perfomed with the alike evolutionary model. TVM + G was the optimal model for *trn*L-F data based on AIC in Modeltest v3.7. Observation of consistency and examined log likelihoods among all independent runs showed that burn-in periods very long enough for chains to become stationary [37]. Figures included nonsignificant bootstrap support (BS) of more than 50% and posterior probabilities of more than 70%.

Median-joining (MJ) network method was effectively employed to study detailed progenitor–descendant relationship among polyploidy species within tribe Triticeae [27, 35, 37, 53]. MJ network analysis was conducted by the Network 4.6.1.3 program (Fluxus Technology Ltd., Clare, Suffolk, UK). For the purpose of preventing single insertion/deletion events from being counted as multiple mutational stages in MJ network study, gaps in aligned nrITS and *trn*L-F sequences were not included in the calculation [37].

## Results

### nrITS data

**Fig. 1:**
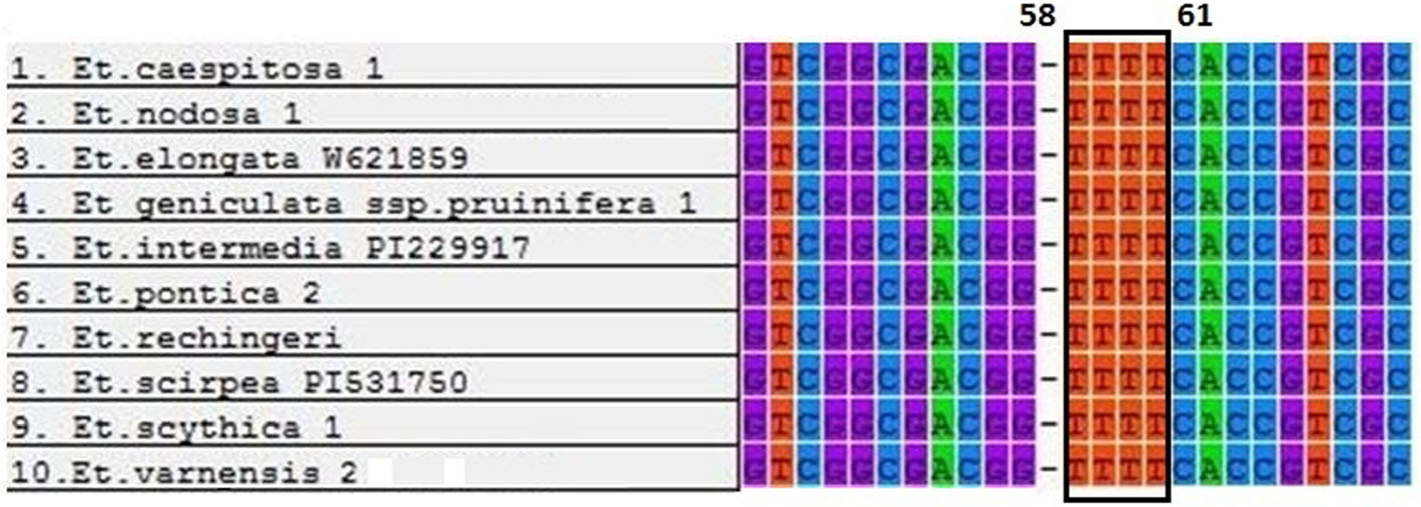
Partial alignment of the amplified sequences of nrITS gene from the ten species of *Elytrigia* sensu lato. A TTTT insert at position 58–61

Comparison of all species analysis suggested that DNA sequences for nrITS ranged from 596 bp to 605 bp in length. A TTTT insert at positions 58–61 in the nrITS sequence was detected for *Et. caespitosa*, *Et. elongata* (W_6_ 21,859), *Et. geniculata* ssp. *pruinifera*, *Et. intermedia* (PI229917), *Et. nodosa*, *Et. pontica*, *Et. rechingeri*, *Et. scirpea* (PI 531750), *Et. scythica*, and *Et. varnensis* (Fig. 1).

**Fig. 2:**
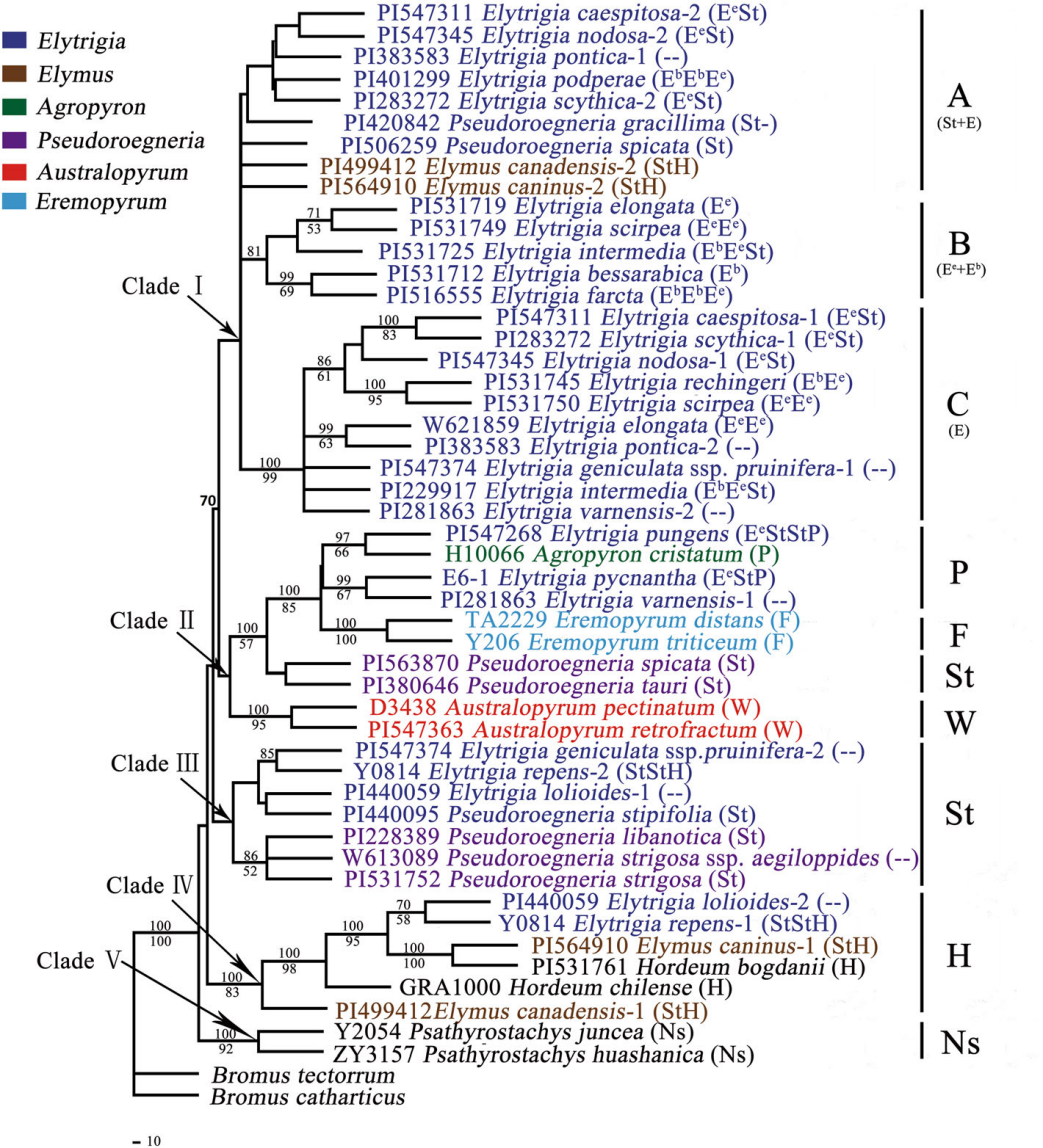
Maximum-likelihood tree (−Lnlikelihood = 2553.6868, base frequencies A: 0.2286, C: 0.2966, G: 0.2794, T: 0.1954, pinvar = none, shape = 0.4121) inferred from the nrITS sequences of *Elytrigia* sensu lato and its affinitive species, under GTR+ G model. Numbers above and below branches indicate posterior probabilities (PP) ≥ 70% by BI analysis and bootstrap support (BS) ≥ 50% by ML, respectively

With assumed nucleotide frequencies A: 0.2286, C: 0.2966, G: 0.2794, and T: 0.1954, nrITS data yielded a single phylogenetic tree (−Lnlikelihood = 2553.6868). Proportion of invariable sites = 0, and gamma shape parameter = 0.4121. Likelihood settings from optimal model (GTR+ G) were selected by AIC in Modeltest v3.7. Similar to that of ML analysis, Bayesian study supposed the same topology. The tree demonstrated in Fig. 2 corresponds to the ML tree with posterior probabilities (PP) above and BS below branches [48].

nrITS region from species were divided into five clades (Clades I–V). Clade I was divided into three groups, namely, A, B, and C. Group A (BS < 50 and PP < 70%) consisted of the St-genome sequence and included two *Pseudoroegneria* species (*Pse. spicata* PI 506259 and *Pse. gracillima*), two *Elymus* species (*El. canadensis* and *El. caninus*), and *Elytrigia* species, such as *Et. caespitosa*, *Et. nodosa*, *Et. podperae*, *Et. pontica*, and *Et. scythica*. Group B (PP = 81%) consisted of *Et. bessarabica*, *Et. elongata* (PI 531719), *Et. farcta* (PI 516555), *Et. intermedia* (PI 531725), and *Et. scirpea* (PI 531749); this group possesses an E-genome sequence. Group C (BS = 99% and PP = 100%) included 10 species with a TTTT insert at positions 58–61; this insert is a possible variation of E-genome sequence. This group comprised *Et. caespitosa*, *Et. elongata* (W_6_ 21,859), *Et. geniculata* ssp. *pruinifera*, *Et. intermedia* (PI 229917), *Et. nodosa*, *Et. pontica*, *Et. rechingeri*, *Et. scirpea* (PI 531750), *Et. scythica*, and *Et. varnensis*. Clade II included St-genome sequences of *Pse. spicata* (PI 563870), *Pse. tauri*, and EStP genome species (*Et. pungens* and *Et. pycnantha*) and unknown genome sequences of *Et. varnensis*, P-genome sequences of *Agropyron cristatum*, W-genome sequences of *Australopyrum pectinatum*, *Au. retrofractum*, and F-genome sequences of *Eremopyrum distans* and *Er. triticeum.* Clade III consisted of St-genome sequences of *Pse. libanotica*, *Pse. stipifolia*, *Pse. strigosa*, *Pse. strigosa* ssp. *aegilopoides*, and three *Elytrigia* s. l. species (*Et. geniculata* ssp. *pruinifera*, *Et. lolioides*, and *Et. repens*). Clade IV comprised two *Elytrigia* s. l. species (*Et. lolioides* and *Et. repens*), two *Elymus* s. l. species (*El. canadensis* and *El. caninus*), and two *Hordeum* s. l. (*H. bogdanii* and *H. chilense*) (BS = 83%; PP = 100%). Clade V comprised *Psathyrostachys juncea* and *Psa. huashanica* (BS = 91% and PP = 100%).

**Fig. 3:**
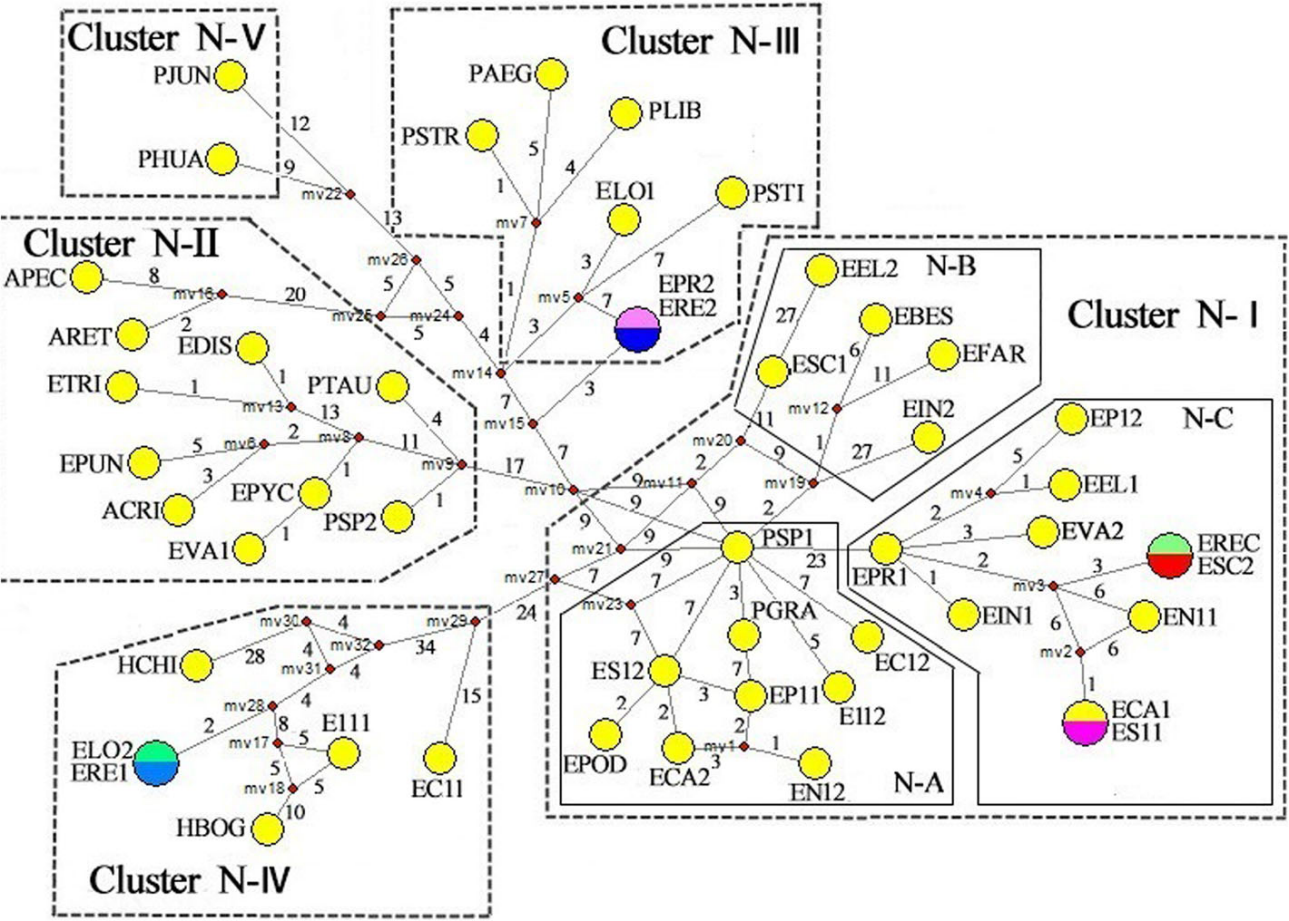
Median-joining networks based on nrITS locus haplotype of *Elytrigia* sensu lato and its affinitive species. Haplotypes are represented by circles. The numbers along the blanches indicate the frequency of mutations. Abbreviations of species names are listed in Table 1

In MJ analysis, each circular network node indicated a single sequence haplotype, and the node size is proportional to the number of isolates with that of haplotype [37]. Median vectors (standing for missing intermediates) present nodes that haven’t sampled deduced by MJ network study, and the numbers along branches illustrate the mutation positions. Distinguishing colors indicated various species species that share similar haplotype circular network node. Either alternative genealogies or true reticulation events are represented by network loops in closely related lineages [37]. The MJ network depicted genealogical relationships among 45 nrITS haplotypes from 49 taxa (Fig. 3) [48]. We found that MJ network represented a consistent phylogenetic reconstruction with ML tree. Then, we determined the names and group names of similar clusters to synchronize the MJ network. In nrITS MJ network analysis, five clusters (Clusters N-I to N-V) formed one star-like radiation. Three clusters (Clusters N-III to N-V) represented three different types of haplotypes (St, P, and Ns types) of *Elytrigia* s. l. and its related genera. Cluster N-I was divided into subclusters N-A, N-B, and N-C with E and St types, and *Pse. spicata* PI 506259 (PSP1) was placed at the central branching point. Cluster N-II included St type of *Pse. spicata* PI 563870 (PSP2) and *Pse. tauri* (PTAU), P type of *Ag. cristatum* (ACRI), *Et. pungens* (EPUN), and *Et. pycnantha* (EPYC), F type of *Er. distans* (EDIS) and *Er. triticeum* (ETRI), W type of *Au. pectinatum* (APEC) and *Au. retrofractum* (ARET), and unknown type of *Et. varnensis* (EVA1).

### *trn*L-F data

**Fig. 4:**
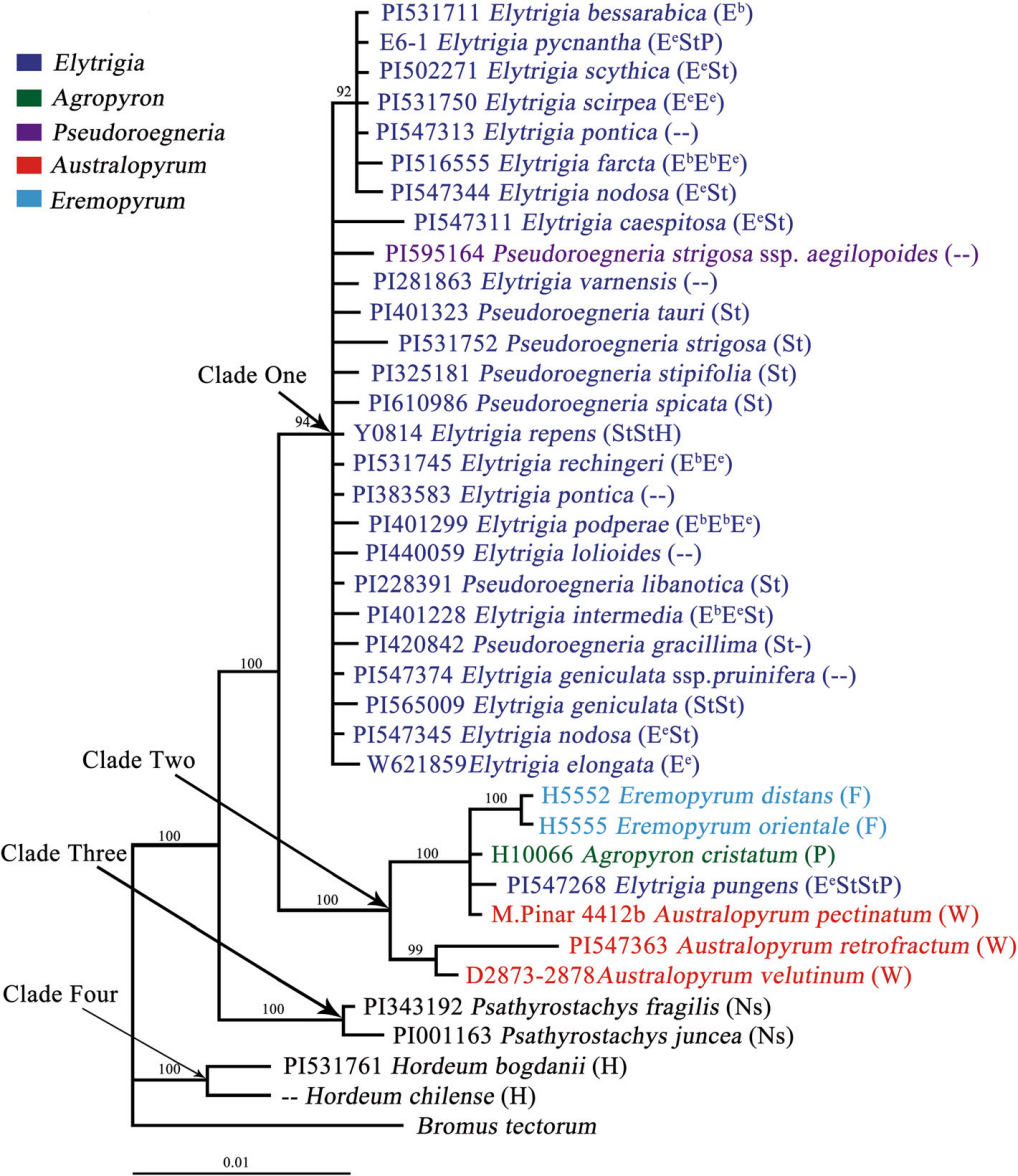
Bayesian inference tree inferred from the *trn*L-F sequences of *Elytrigia* sensu lato and its affinitive species. Numbers above branches indicate posterior probabilities (PP) ≥ 70% by BI

Comparison of all species studies showed that the length of *trn*L-F sequences ranged from 809 bp to 882 bp. Likelihood settings from optimal model (TVM + G) were chosed by AIC in Modeltest v3.7. Fig. 4 illustrates the BI tree with PP above branches. All *trn*L-F sequences from *Elytrigia* and its related genera species were similar.

Clade One involved 17 *Elytrigia* s. l. species [*Et. bessarabica*, *Et. caespitosa*, *Et. elongata*, *Et. farcta*, *Et. geniculata*, *Et. geniculata* ssp. *pruinifera*, *Et. intermedia*, *Et. lolioides*, *Et. nodosa* (PI 547344 and PI 547345), *Et. podperae*, *Et. pontica* (PI 383583 and PI 547313), *Et. pycnantha*, *Et. rechingeri*, *Et. repens*, *Et. scirpea*, *Et. scythica*, and *Et. varnensis*] and seven *Pseudoroegneria* species (*Pse. gracillima*, *Pse. libanotica*, *Pse. spicata*, *Pse. stipifolia*, *Pse. strigosa*, *Pse. strigosa* ssp. *aegilopoides*, and *Pse. tauri*). All diploid species with F, P, and W genomes were clustered together in Clade Two. Ns type *trn*L-F sequences from *Psa. fragilis* and *Psa. juncea* and H type *trn*L-F sequences from *H. bogdanii* and *H. chilense* were placed at Clusters Three and Four, respectively.

**Fig. 5:**
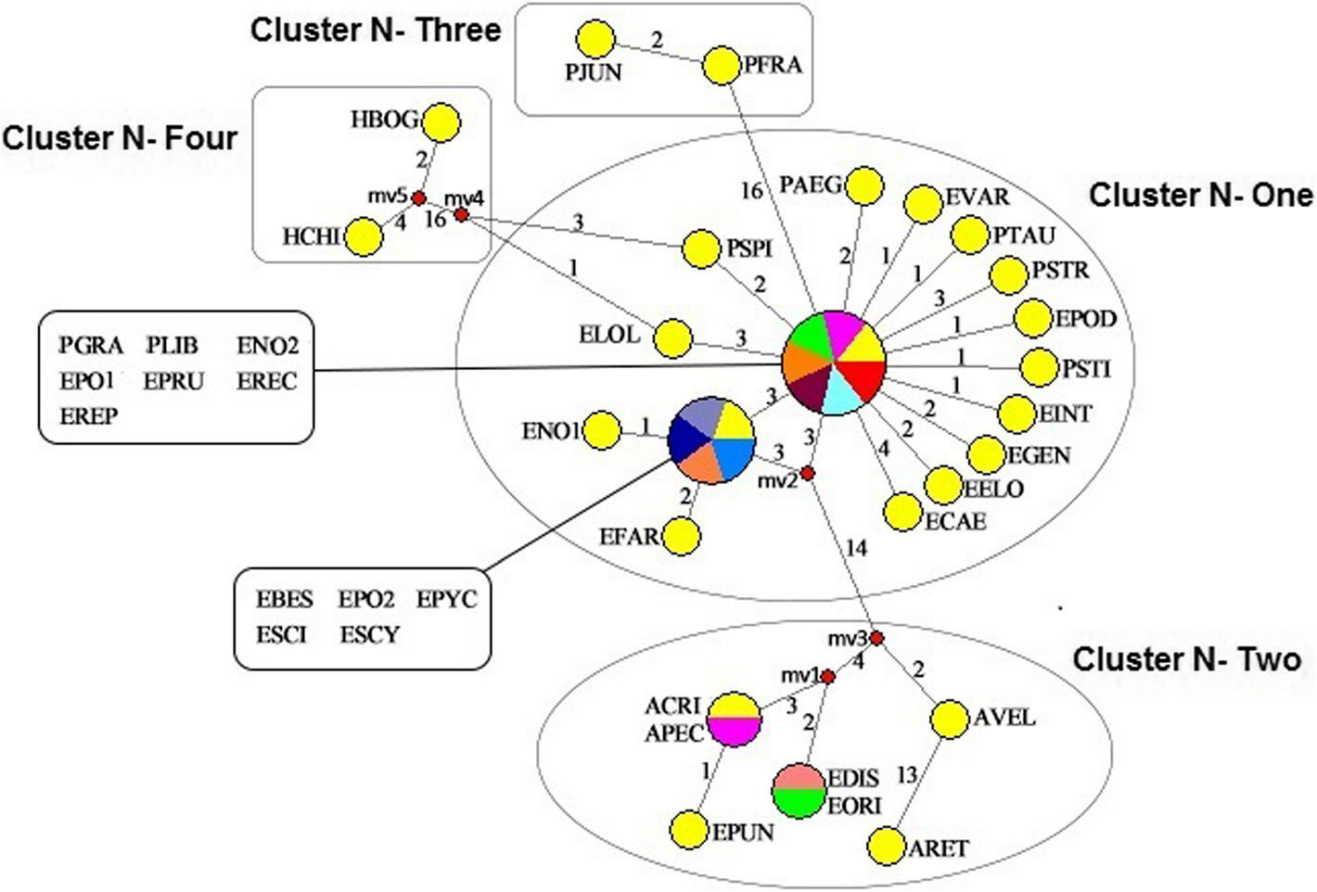
Median-joining networks based on *trn*L-F locus haplotype of *Elytrigia* sensu lato and its affinitive species. Haplotypes are represented by circles. The numbers along the blanches indicate the frequency of mutations. Abbreviations of species names are listed in Table 1

In the *trn*L-F MJ network analysis, 25 haplotypes were derived from 37 taxa. MJ network represented consistent phylogenetic reconstruction with BI tree. We determined clusters’ names following the name of groups shown in the ML tree. The *trn*L-F MJ network was divided into four clusters (Clusters N-One to N-Four). All species containing E or St genome were clustered together with E or St diploid species in Cluster N-One. Cluster N-Two included F, P, and W types of haplotypes. Ns type of *Psathyrostachys* haplotype species and H type of *Hordeum* haplotype species were grouped, respectively, in Clusters N-Three and N-Four (Fig. 5).

## Discussion

### Phylogenetic relationships among species in *Elytrigia* s. l.

*Elytrigia* s. l. is distributed in subtropical and warm temperate regions of both hemispheres [6]. Classification and systematic position of *Elytrigia* remain controversial [7, 8, 54–56]. Traditionally, the classification based on morphology and *Elytrigia* species contains E^e^, E^b^, E^e^E^b^, E^e^E^e^St, E^b^E^e^St, E^e^St, StSt, StH, and EStP genomes. However, Dewey [1] and Löve [8] showed that taxonomic treatment of Triticeae species should be depended on genomic constitution. Therefore, *Elytrigia* species must be reclassified. Several current studies used molecular biology to study phylogenetic relationships of *Elytrigia* s. l. species and its related genera [43, 57, 58]. Hsiao et al. [59] estimated phylogenetic relationships of 30 diploid species of Triticeae (Poaceae) from the nrITS region of nuclear ribosomal DNA. Results illustrated that each genome group of species is monophyletic and consisten with cytogenetic evidence, and *Australopyrum* (Tzvelev) A. Löve (W) is closely concerned with *Agropyron* Gaertn. (P) [59]. Kim et al. [43] analyzed nrITS haplotypes, revealing close relationship of E, P, and St genomes. Cytologically, St and Y genomes and St, P, and W genomes are closely related [2, 60–62]. This finding indicates close relationship of E, P, St, and W genomes.

In this study, based on nrITS data, all *Elytrigia* s. l. species were classified in four groups (E, H, P, and St types) in the ML tree and MJ network. These results indicated that *Elytrigia* s. l. species contain different genomes. These findings also strongly support previous results. Genome species are not highly supported in Clades I-B, E^e^, and E^b^ (BS < 50% and BI = 81%) (Fig. 2). This phenomenon provides evidence of close affinity between E^e^ and E^b^ genome species. Thus, these species are not homologous but homoeologous [63]. Our phylogenetic results also support previous cytological investigations reported by Löve [8, 64, 65], Yen and Yang [3], and Zhou [11].

### Phylogenetic relationships between *Elytrigia* s. l. and related genera

In the present study, in the ML tree and MJ network based on nrITS data, seven types of nrITS region (E, F, H, P, W, Ns, and St types) were obtained from the *Elytrigia* s. l. species and its related genera. In polyploidy species *Et. repens* (StStH), H type was clustered with *Hordeum* species in Clade IV (83% BS, 100% PP), and St type was clustered with *Pseudoroegneria* (Fig. 2). In this study, we failed to obtain E and St type nrITS sequences from *Et. pycnantha* (EStP) and *Et. pungens* (EStP), whereas P type was clustered with *Agropyron* in Clade II (86% BS and 97% PP). In the phylogenetic tree, Clades II and IV formed a monophyletic group, and results support the distant relationship between H genome and other genomes (E, P, St, and W) (Fig. 2). This finding also indicated that St genome is the origin of *Pseudoroegneria*, whereas P genome is the origin of *Agropyron.*

*Eremopyrum* (Ledeb.) Jaub. et Spach and *Agropyron*. Gaertn. are highly similar based on one-keeled glumes and caryopsis morphology [66, 67]. According to molecular phylogenetic analysis, *Er. triticeum* and *Er. distans* were clustered with *Agropyron* based on *rpo*A, cpDNA, *DMC*1, and *β*-amylase data [44, 68–70]. Fan et al. [26] showed that *Eremopyrum* and *Agropyron* are closely related based on the presence of *Acc*1, *Pgk*1, and *Acc*1 + *Pgk*1. In the present nrITS gene data, allopolyploid species of *Et*. *pycnantha* and *Et*. *pungens* (EStP) were clustered with *Er*. *triticeum*, *Er*. *distans*, and *Ag. cristatum* with high statistical support (85% BS and 100% PP). *Et*. *pycnantha*, *Et*. *pungens*, *Eremopyrum*, and *Agropyron* species were grouped with St genome diploid species (*Pse. spicata* PI563870 and *Pse. tauri*) (57% BS and 100% PP) (Fig. 2). Estimates strongly support that *Eremopyrum* and *Agropyron* are closely related, and St, P, and F are very close to each other.

Sha et al. [48] studied phylogenetic relationships of *Leymus* based on *trn*H*-psb*A and indicated that *Pseudoroegneria* species are close to *Lophopyrum bessarabicum.* Evidence from meiotic chromosome pairing [71], morphological data [72], and DNA sequencing [44] suggested close relationship among *Lo. bessarabicum*, *Lo. elongatum*, and *Pseudoroegneria*. In the BI tree and MJ network based on *trn*L-F sequences, all *Elytrigia* species were categorized under Clade One with a number of zero-length branches; *Elytrigia* species are sisters with diploid species of *Eremopyrum* (F), *Agropyron* (P), and *Australopyrum* (W) (94% PP). H and Ns genome species formed the monophyletic group. These results indicated minimal differences in E and St genomes based on *trn*L-F sequence and close relationship of E, F, P, St, and W genomes, which are distant from H and Ns genomes. These findings support previous studies on morphology [66, 67], molecular biology [5, 44, 68–70], and cytogenetics [2, 60, 61].

### Putative maternal donor and origin of *Elytrigia* species

cpDNA is mostly inherited from the female parent in tall plants. Therefore, it can be used to determine maternal donor in polyploids [37, 53, 73]. In the *trn*L-F ML, the phylogenetic tree showed high numbers of zero-length branches, which are mainly related to multifurcating relationships. Tree-based study methods are unable to represent multifurcating relationships and the coexistence of ancestors with their derivatives [53, 74]. Network approaches were designed to deal with such multifurcations [53, 73–76].

Previous studies based on cpDNA indicated that *Pseudoroegneria* (St genome donor) species are the maternal donor for species of Triticeae [41, 44, 77]. However, cytologically, Yen et al. [78] considered that rather than the St genome, the maternal donor of *Kengyilia* is the origin of P genome species. In this *trn*L-F-based BI tree, *Et. bessarabica* (2×) was clustered with polyploidy species *Et. farcta*, *Et. nodosa* (PI 547344), *Et. pontica* (PI 547313), *Et. pycnantha*, *Et. scirpea*, and *Et. scythica* (92% PP). MJ network analysis showed that diploid species *Et. bessarabica* (E), *Et. pontica* (PI 547313), *Et. pycnantha* (EStP), *Et. scirpea* (E), and *Et. scythica* (ESt) exhibit the same haplotype in Cluster N-One (Fig. 5). Combined with BI and MJ analyses, we can conclude that E genome-diploid species in *Elytrigia* served as maternal donor of E genome for *Et. farcta*, *Et. nodosa* (PI 547344), *Et. pontica*, *Et. pycnantha*, and *Et. scythica*. This conclusion agrees with results of Liao et al. [37]. *Et. nodosa* (P I547345 Ukraine) was not clustered with *Et. bessarabica*, and its haplotype differs from that of *Et. nodosa* (PI 547344 Turkey). Results showed that (1) at no less than two species served as maternal donor, indicating that formation of *Et. nodosa* occur multiple times. A similar conclusion was observed based on *Et. caespitosa*, *Et. intermedia*, *Et. varnensis*, and *Kengyilia* species [45, 58, 79]. (2) Different maternal donors in *Et. nodosa* are affected by altitude and climate conditions [80]. In the BI tree based on *trn*L-F sequence, we can conclude that *Pseudoroegneria* species (St genome donor) acted as maternal donor of *Et. repens* (StStH), whereas species of *Agropyron* Gaertn. (P genome donor) acted as maternal donor of *Et. pungens* (EStStP). However, E genome acted as maternal donor of *Et. pycnantha* (EStP). This result indicated that different species served as maternal donors that contributed to species containing the same genomes. Previous findings on *Et. intermedia* were similar to our results [58]. Other polyploidy species in *Elytrigia* and diploid species containing E or St genome formed zero-length branches in Clade One because of the close relation of E and St genomes (Fig. 4). Sources of maternal donor of these genomes remain to be identified.

### Differentiation and relationship between E and St genomes

In Clade I, species containing E, St, and ESt genomes and those in Cluster N-I *Pse. spicata* appeared at the central part, indicating close relationship of St and E genome species (Figs. 2 and 3). These findings coincide with previous findings on morphology and molecular biology [44, 72, 81]. In the present study, E and St types were obtained from species containing ESt genome grouped with *Elytrigia* or *Pseudoroegneria* diploid species, respectively. This phenomenon showed that E genome was the origin of diploid *Elytrigia* species with the E genome. St genome was the origin of *Pseudoroegneria.* Results from morphology, genetics, and molecular biology indicate that species containing E, St, and ESt genomes are closely related with *Elytrigia*.

### Taxonomy of species with ESt and EStP genomes

Polyploidization and hybridization are long recognized as prominent forces in evolution of plant species, which feature consequences of genomic changes [22, 23]. Genome relationship and differentiation are often vague in some species because of frequent introgression of alien genes, polyploidization and chromosome segments from wide hybridization [43]. Thus, classification is one of the most important issues that require understanding.

Previous studies indicated that *Et. caespitosa*, *Et. intermedia*, *Et. nodosa*, *Et. scythica*, and *Et. varnensis* contain ESt genomes, which belong to *Trichopyrum* [3, 17, 82, 83]. Comparison of partial sequences of nrITS gene showed that a TTTT insert at positions 58–61 in nrITS sequence was detected for 11 species (Fig. 1). This finding indicated that introgression of E genome during polyploidization or different independent hybridization events may create the variants in polyploidy ESt species. In the ML tree and MJ network based on nrITS sequence, one group is formed by ESt genome species (*Et. caespitosa*, *Et. intermedia*, *Et. nodosa*, *Et. scythica*, and *Et. geniculata* ssp. *pruinifera*) and unknown genome species of *Et. varnensis*. This result indicated that these species should be classified under the same genus (*Trichopyrum*). Species containing ESt genomes were grouped with diploid species *Et. elongata* (E genome), suggesting that E genome may be derived from *Et. elongata*. In this study, diploid species of *Et. elongata* were differentiated. We selected two *Et. elongata* (Iran, France) with different origins, which are divided into Clades I-B and C (Fig. 2). A TTTT insert at positions 58–61 in the sequence was also detected for *Et. elongata* (W_6_ 21,859) (Fig. 1). This result indicates that ESt genome polyploid species and diploid *Elytrigia* species (E genome) displayed hybridization event, resulting in divided E genome.

*Et. varnensis* was reported by Löve to contain ESt genomes (2n = 12× = 84) [8]. Yang [84] showed that *Et. varnensis* is a tetraploid species. Diversity of species ploidy may be caused by chromosome variation under natural conditions. In this study, we discovered that *Et. varnensis* clustered with *Et. pungens*, *Et. pycnantha*, *Ag. cristatum*, *Au. pectinatum*, *Au. retrofractum*, *Er. distans*, and *Er. triticeum* (85% BS and 100% PP) (Fig. 2)*.* We concluded that this species contains P or F genome. Another estimate indicated that St genome allopolyploid species possibly resulted from introgression of *Eremopyrum* or *Agropyron* during polyploidization. Results strongly support those of previous studies in cytogenetics [85].

### Possible genome constitutions and taxonomic treatment of *Et. lolioides*

Cytologically, *Et. repens* comprised StH genome. *Et. lolioides* is a polyploid species, and its genomic constitutions remains unknown [8, 17, 62, 86]. In the present study, *Et. lolioides* was clustered with diploid *Pse. libanotica* (St genome); this result indicated that *Et. lolioides* possesses one St genome. *Et. lolioides* clustered with *H. bogdanii*, *H. chilense* (H), and *Et. repens* (H copy) with high statistical result (98% BS and 100% PP) (Fig. 2). Such finding also indicated that *Et. lolioides* contains H genome and is closely related to StH genome species *Et. repens*. Thus, we can conclude that genomic constitution of *Et. lolioides* includes St and H genomes and belongs to *Elymus* s. l.

## Conclusion

This study analyzed the phylogenetic relationship among *Elytrigia* s. l. species on the basis of the nrITS sequence data. The results supported the conclusion that *Elytrigia* s. l. consists of various genomes (E, H, P, and St types), which should be classified as different genera. Analyses based on nrITS sequence data and chloroplast *trn*L-F region show that the E, F, St, P, and W genomes have a close intergroup relationship but are distant with the H and Ns genomes. This finding strongly supports previous studies on morphology, molecular biology, and cytogenetics. nrlTS sequence analysis demonstrated that the E genome of species *Et. caespitosa*, *Et. caespitosa* ssp*. nodosa*, *Et. intermedia*, *Et. scythica* and *Et. geniculata* ssp. *pruinifera*, which contains ESt genomes, originated from *Et. elongata* in *Lophopyrum*. However, differentiation was found in diploid species *Et. elongata*; this phenomenon was possibly due to diverse geographical origins or introgression. *Et. lolioides*, which is composed of unknown genomes, contains the H and St genomes and has a close genetic relationship with *Et. repens* and *El. canadensis*, which contain the St and H genomes. Accordingly, the genome of *Et. lolioides* is inferred to contain St and H. In this paper, polyploid species of *Elytrigia* s. l. was deduced based on *trn*L-F sequence, the female parent of *Et. caespitosa* ssp. *nodosa* (PI547344), *Et. farcta, Et. pontica* (PI547313), *Et. pycnantha*, *Et. scirpea* and *Et. scythica* is the diploid species of *Elytrigia* s. l. containing the E genome; the maternal donor of the polyploidy species *Et. caespitosa* ssp. *nodosa* (PI547345), *Et. pontica* (PI383583), *Et. repens, Et. geniculata* ssp. *pruinifera* is the St genome. Different maternal donors were also found in allopolyploid species. This result could be attributed to different growth environments, introgression, or incomplete separation of genome E lineage. Thus, different haplotypes were presented.

## Abbreviations

AIC: Akaike information criterion
BI: Bayesian inference
BS: Bootstrap support
CTAB: Cetyltrimethyl ammonium bromide
MJ: Median-joining
ML: Maximum likelihood
nrITS: Nuclear ribosomal internal transcribed spacer
PP: Posterior probabilities
